# nNOS(+) striatal neurons, a subpopulation spared in Huntington's Disease, possess functional NMDA receptors but fail to generate mitochondrial ROS in response to an excitotoxic challenge

**DOI:** 10.3389/fphys.2013.00112

**Published:** 2013-05-16

**Authors:** Lorella M. T. Canzoniero, Alberto Granzotto, Dorothy M. Turetsky, Dennis W. Choi, Laura L. Dugan, Stefano L. Sensi

**Affiliations:** ^1^Department of Biological and Environmental Science, University of SannioBenevento, Italy; ^2^Molecular Neurology Unit, Center of Excellence on Aging, University “G. d'Annunzio”Chieti, Italy; ^3^Department of Biochemistry and Microbiology, Oklahoma State UniversityTulsa, OK, USA; ^4^Department of Neurology, Stony Brook UniversityStony Brook, NY, USA; ^5^Department of Medicine, University of California San DiegoSan Diego, CA, USA; ^6^Department of Neuroscience, University of California San DiegoSan Diego, CA, USA; ^7^Department of Neuroscience and Imaging, University “G. d'Annunzio”Chieti, Italy; ^8^Departments of Neurology and Pharmacology, Institute for Memory Impairments and Neurological Disorders, University of California IrvineIrvine, CA, USA

**Keywords:** NADPH diaphorase, excitotoxicity, reactive oxygen species, nitric oxide synthase, Huntington's disease

## Abstract

Huntington's disease (HD) is a neurodegenerative condition characterized by severe neuronal loss in the cortex and striatum that leads to motor and behavioral deficits. Excitotoxicity is thought to be involved in HD and several studies have indicated that NMDA receptor (NMDAR) overactivation can play a role in the selective neuronal loss found in HD. Interestingly, a small subset of striatal neurons (less than 1% of the overall population) is found to be spared in post-mortem HD brains. These neurons are medium-sized aspiny interneurons that highly express the neuronal isoform of nitric oxide synthase (nNOS). Intriguingly, neurons expressing large amounts of nNOS [hereafter indicated as nNOS(+) neurons] show reduced vulnerability to NMDAR-mediated excitotoxicity. Mechanisms underlying this reduced vulnerability are still largely unknown and may shed some light on pathogenic mechanisms involved in HD. One untested possibility is that nNOS(+) neurons possess fewer or less functioning NMDARs. Employing single cell calcium imaging we challenged this hypothesis and found that cultured striatal nNOS(+) neurons show NMDAR-evoked responses that are identical to the ones observed in the overall population of neurons that express lower levels of nNOS [nNOS(−) neurons]. NMDAR-dependent deregulation of intraneuronal Ca^2+^ is known to generate high levels of reactive oxygen species of mitochondrial origin (mt-ROS), a crucial step in the excitotoxic cascade. With confocal imaging and dihydrorhodamine (DHR; a ROS-sensitive probe) we compared mt-ROS levels generated by NMDAR activation in nNOS(+) and (−) cultured striatal neurons. DHR experiments revealed that nNOS(+) neurons failed to produce significant amounts of mt-ROS in response to NMDA exposure, thereby providing a potential mechanism for their reduced vulnerability to excitotoxicity and decreased vulnerability in HD.

## Introduction

Excitotoxicity is a major pathogenic component of several neurodegenerative disorders, including Alzheimer's disease, Parkinson's disease, amyotrophic lateral sclerosis, and Huntington's disease (HD) (Choi, 2005; Lau and Tymianski, 2010; Spalloni et al., 2013). HD is an autosomal dominant neurodegenerative condition characterized by severe behavioral, cognitive, and movement disorders (Ross and Tabrizi, 2011). Inheritance of the huntingtin (Htt) protein showing a pathogenic expansion of a glutamine stretch (polyQ repeats >35) (Macdonald et al., 1993) leads to massive cortical and striatal neuronal loss (Halliday et al., 1998; Cattaneo et al., 2005; Guo et al., 2012). Reasons for this sub-regional selectivity of the neurodegenerative process are not completely understood, although several mechanisms have been proposed. In that respect, evidence indicates that polyQ Htt promotes HD pathology through deregulation of vesicle trafficking (Difiglia et al., 1995; Qin et al., 2004), alteration of BDNF transport (Gauthier et al., 2004), disruption of microtubules (Trushina et al., 2003), interference with NMDA receptor (NMDAR) and synaptic activity (Zeron et al., 2004) as well as disruption of mitochondrial functioning and morphology (Rockabrand et al., 2007; Costa and Scorrano, 2012).

Excitotoxicity is a phenomenon driven by excessive synaptic accumulation of glutamate and associated with dysregulation of intraneuronal Ca^2+^ ([Ca^2+^]_i_) homeostasis (Choi, 2005). A major feature of excitotoxicity is the NMDAR/Ca^2+^-dependent enhanced generation of nitric oxide (NO) and other reactive oxygen species (ROS) [reviewed in Forder and Tymianski (2009) and Szydlowska and Tymianski (2010)].

A subpopulation of striatal neurons (accounting for less than 1% of the overall population) expresses high levels of the enzyme nicotinamide adenine dinucleotide phosphate-diaphorase (NADPH-d) that is the neuronal isoform of nitric oxide synthase (nNOS) (Hope et al., 1991). Intriguingly, these neurons expressing large amount of nNOS [hereafter called nNOS(+) neurons] are not affected by NMDAR-dependent toxicity and spared in the striatum of HD patients (Ferrante et al., 1985; Koh et al., 1986; Koh and Choi, 1988). The molecular and biochemical determinants of this decreased vulnerability are still not completely known. A possible simple explanation for the phenomenon is that nNOS(+) neurons have fewer or less active NMDARs. To test this hypothesis, we employed single cell Ca^2+^ imaging and assessed differences in NMDAR-evoked rises in [Ca^2+^]_i_ in striatal cultures, and compared responses obtained in nNOS(+) and the overall population of striatal neurons that express lower levels of the enzyme and hereafter called nNOS(−) neurons.

Mitochondrial function is critical to maintaining [Ca^2+^]_i_ homeostasis (Pizzo et al., 2012). Mutant Htt promotes mitochondrial dysfunction and we have previously shown that specific Htt domains are crucial to drive its mitochondrial localization and promote dysfunction (Rockabrand et al., 2007).

Ca^2+^ overload in mitochondria promotes generation of free radicals in the organelles (Dugan et al., 1995; Reynolds and Hastings, 1995), a step that has been shown to be instrumental in the initiation of the excitotoxic cascade (White and Reynolds, 1995, 1996; Nicholls and Budd, 1998; Stout et al., 1998; Nicholls and Budd, 2000; Nicholls and Ward, 2000; Votyakova and Reynolds, 2001; Nicholls, 2002, 2004; Nicholls et al., 2003, 2007; Rintoul et al., 2003; Reynolds et al., 2004; Vesce et al., 2004; Votyakova and Reynolds, 2005). Thus, we also tested whether exposure to NMDA in striatal cultures generated different levels of mitochondrial ROS (mt-ROS) in nNOS(+) neurons compared to the overall population of nNOS(−) cells.

## Materials and methods

### Chemicals

Tissue culture media and sera were purchased from GIBCO (Life Technologies). Fluorescent calcium (fura-2 AM, and fluo-4FF AM) and ROS (dihydrorhodamine, DHR) indicators were purchased from Molecular Probes (Life Technologies). All other chemicals, unless otherwise specified, were purchased from Sigma-Aldrich.

### Neuronal striatal cultures

All the procedures involving animals were approved by the institutional Ethics Committee (Ce.S.I.) and performed accordingly to institutional guidelines and in compliance with national and international laws and policies.

Striatal cell cultures were prepared from fetal (E15 or E16) Swiss–Webster or CD1 mice. Striatal tissues were dissected in ice-cold dissecting medium and then placed in trypsin (0.25%) for 10 min at 37°C. Tissues were centrifuged, supernatant discarded, and pellet mechanically dissociated with a glass Pasteur pipette. Cells were then resuspended in plating medium containing either: (for mixed cultures) Eagle's Minimal Essential Medium (with 20 mM glucose, 26.2 mM NaHCO_3_) supplemented with L-glutamine (2 mM), 5% fetal calf serum, and 5% horse serum (Hyclone), or (for near-pure neuronal cultures) Neurobasal Medium supplemented with L-Glutamine (0.5 mM), 5% fetal bovine serum, 5% horse serum, 1× B27, and 0.2% penicillin/streptomycin.

To prepare mixed cultures, cell suspensions were diluted and plated onto an astrocytes layer on 35 mm culture dishes with a glass bottom (Mat-Tek). Cells were fed twice a week with a growth medium (containing 10% horse serum, and 2 mM L-glutamine) and after 12 days *in vitro* (DIV) with a serum-free medium supplemented with 2 mM L-glutamine.

For near-pure neuronal cultures, cells suspensions were diluted and plated onto laminin/poly-DL-lysine coated glass coverslips. Three days after plating, non-neuronal cell growth was inhibited by adding 10 μM of cytosine arabinofuranoside. Twice a week, 25% of the medium was replaced with equal amounts of fresh Neurobasal medium.

Striatal neurons were used between 12 to 17 DIV.

### Imaging studies

Ca^2+^ imaging employing fura-2 was performed using a Nikon Diaphot inverted microscope equipped with a Xenon lamp, a 40× Nikon epifluorescence oil immersion objective (N.A.: 1.3), and a CCD camera (Quantex). Fluo-4FF experiments were instead performed using a Nikon Eclipse TE300 inverted microscope equipped with a Xenon lamp, a 40× Nikon epifluorescence oil immersion objective (N.A.: 1.3) and a 12-bit Orca CCD camera (Hamamatsu). DHR experiments were performed with a confocal microscope (Noran Odyssey) equipped with an argon-ion laser, an inverted microscope (Nikon Diaphot), and a 60× Nikon oil-immersion objective (N.A.: 1.4). Fura-2 ratios and DHR confocal images (and relative bright field images) were digitized and analyzed using Image-1 system (Universal Imaging) or Metamorph imaging software (Universal Imaging), respectively. Fluo-4FF images were acquired and analyzed with Metafluor 6.0 software (Molecular Devices).

#### [Ca^2+^]_i_ measurements

Striatal cultures were loaded for 30 min in the dark with fura-2 AM (5 μM) or fluo-4FF AM (5 μM) plus 0.2% Pluronic F-127 in HEPES-buffered saline solution (HCSS) (120 mM NaCl, 5.4 mM KCl, 0.8 mM MgCl_2_, 20 mM HEPES, 15 mM glucose, 1.8 mM CaCl_2_, 10 mM NaOH, pH 7.4), washed, and incubated for further 30 min in HCSS. In fura-2 experiments [Ca^2+^]_i_ was determined using the ratio method described by Grynkiewicz et al. (1985). Fura-2 (Ex λ: 340, 380 nm, Em λ: 510 nm) calibrated values were obtained by determining *R*_min_ and *R*_max_ using: EGTA (10 mM) and ionomycin (10 μM) in 0 Ca^2+^ buffer for *R*_min_, and Ca^2+^ (10 mM) with ionomycin (10 μM) for *R*_max_. Fura-2 *K*_*d*_ was set at 225 nM. Results are reported as mean [Ca^2+^]_i_ nM ± SEM. In fluo-4FF (Ex λ: 490 nm, Em λ: 510 nm) fluorescence changes of each cell (*F*_*x*_) were normalized to basal fluorescence intensity (*F*_0_). Results are expressed as mean *F*_*x*_/*F*_0_ ± SEM values. In all experiments, NMDA (50 μM + 10 μM glycine) was applied for 20 s and then removed through a rapid perfusion system.

#### ROS production measurements

Cells were loaded with DHR (5 μM) in the dark in a 37°C/5% CO_2_ incubator for 30 min and then studied with confocal imaging. DHR was excited at 488 nm and emission collected at >515 nm. In order to minimize DHR photo-oxidation, laser beam was used to less than 5% of full power and image acquisition intervals minimized to ≤ 2 s every 5 min. DHR was maintained in the buffer throughout all the imaging session to maintain probe equilibration between the inside and outside of cells.

NMDA exposure was performed by adding NMDA (50 μM + 10 μM glycine) to the baseline HCSS solution for 5 min. NMDARs activation was then halted by addition of 10 μM MK-801 and neurons imaged for additional 25 min.

### NADPH-diaphorase staining

To identify what we call nNOS(+) neurons we employed the NADPH-d staining procedure (Koh et al., 1986). To that aim, after Ca^2+^_i_ or DHR experiments, cultures were rinsed three times in ice-cold TBS and fixed for 30 min at 4°C in 4% paraformaldehyde/0.1 M PBS buffer. After fixation, dishes were washed with TBS and staining solution applied for 1 h at 37°C. NADPH-d staining solution contained: 0.1 mM Tris/HCl, 0.2% Triton X-100, 1.2 mM sodium azide, 0.2 mM nitrotetrazolium blue, and 1 mM NADPH, pH 7.2. The staining solution was removed and cultures rinsed with TBS. After staining, dishes were re-inserted in the microscope stage and fields re-matched with those previously imaged with fura-2, fluo-4FF, and DHR. nNOS(+) neurons identified as NADPH-d (+) under bright-field illumination were then evaluated for their responses in the imaging experiments.

### Statistical analysis

Grubbs' test was performed to detect outliers, the significance level was set at α = 0.05 (no nNOS(+) were found to be significant outliers). Statistical analysis was performed using the Student's *t*-test for unpaired data. Results were considered statistically significant at *p* < 0.05.

## Results

### [Ca^2+^]_i_ rises upon NMDA exposure in nNOS(+) and (−) striatal neurons

In this set of experiment, we tested whether nNOS(+) possess functional NMDARs and evaluated NMDAR-dependent [Ca^2+^]_i_ increases as an indirect parameter of receptor activity. [Ca^2+^]_i_ rises upon NMDA exposure were investigated with single cell Ca^2+^ imaging. This indirect assay is the only possible way to study NMDAR activity in specific nNOS(+) neurons. A more direct approach would have been to investigate NMDAR-evoked currents with patch clamp electrophysiology. Unfortunately, this approach is highly unfeasible given the extremely low density (<1%) of nNOS(+) neurons in our striatal cultures along with the absence of any suitable marker to identify these neurons when in culture, two factors making very unlikely the possibility of successfully patching on to these cells in adequate numbers.

**Figure 1:**
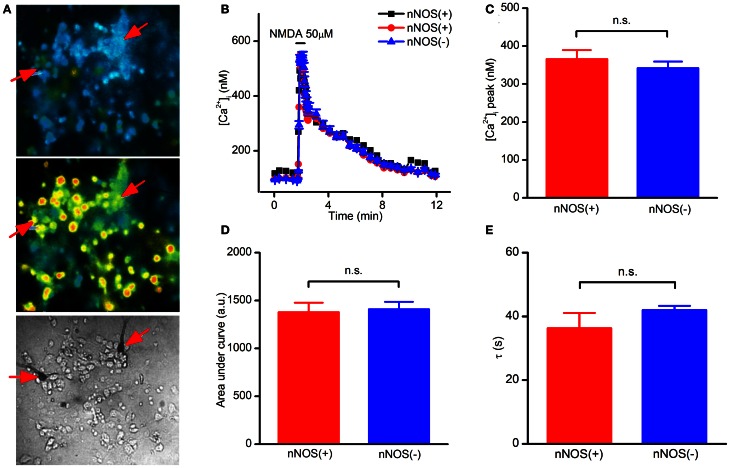
**NMDAR-dependent [Ca^2+^]_i_ rises in striatal cultured neurons evaluated with a high affinity Ca^2+^-sensitive probe. (A)** Fluorescence images of fura-2 loaded striatal neurons before (top), and upon NMDA exposure (middle). Bright field image (bottom) showing stained nNOS(+), indicated by arrows, and nNOS(−)neurons. **(B)** Time course of NMDAR-dependent [Ca^2+^]_i_ rises, traces of a single experiment [NOS(+) (*n* = 2) and nNOS(−) (*n* = 47)], representative of 20 independent experiments. **(C)** Average [Ca^2+^]_i_ peak amplitude in the two neuronal populations (*p* = 0.42). **(D)** [Ca^2+^]_i_ dynamics expressed as area under the curve (*p* = 0.70). **(E)** Recovery phase following NMDA exposure (*p* = 0.43). Results are expressed as mean values ± SEM; nNOS(+) *n* = 22; nNOS(−) *n* = 560.

Striatal cultures loaded with fura-2, a high affinity Ca^2+^ probe (*K*_*d*_ = 225 nM), were exposed to NMDA (50 μM + 10 μM glycine) and [Ca^2+^]_i_ elevation assessed during and after the challenge. In this set of experiments, we observed that NMDAR-dependent [Ca^2+^]_i_ rises occurring in nNOS(+) were not statistically different from those found in the overall population of nNOS(−) neurons (Figures 1A,B). To dissect and possibly reveal more subtle differences in [Ca^2+^]_i_ handling between nNOS(+) and (−) neurons, we analyzed peak amplitudes, areas under the curve (an index of the overall cytosolic Ca^2+^ load) and recovery phase time (τ) of the [Ca^2+^]_i_ changes (Figures 1C–E). None of these parameters showed statistically significant differences between the two neuronal populations. Analysis of baseline [Ca^2+^]_i_ levels also showed no differences between nNOS(+) and (−) neurons at rest (data not shown).

**Figure 2:**
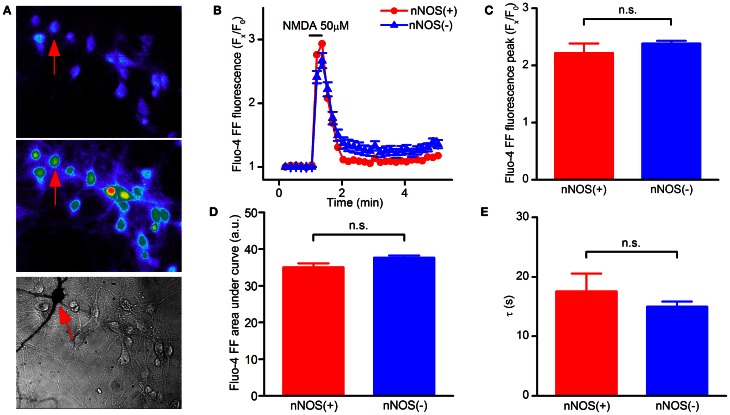
**NMDAR-dependent [Ca^2+^]_i_ rises in striatal cultured neurons evaluated with a low affinity Ca^2+^-sensitive probe. (A)** Fluorescence images of fluo4-FF loaded striatal neurons before (top) and upon NMDA exposure (middle). Bright field image (bottom) showing stained nNOS(+), indicated by arrows, and nNOS(−) neurons. **(B)** Time course of NMDAR-dependent [Ca^2+^]_i_ rises, traces of a single experiment [nNOS(+) (*n* = 1) and nNOS(−) (*n* = 18)], representative of 12 independent experiments. **(C)** Average Ca^2+^_i_ peak amplitude in the two neuronal populations (*p* = 0.36). **(D)** [Ca^2+^]_i_ dynamics expressed as area under the curve (*p* = 0.24). **(E)** Recovery phase following NMDA exposure (*p* = 0.43). Results are expressed as mean values ± SEM; nNOS(+) *n* = 13; nNOS(−) *n* = 165.

In the fura-2 data set we noticed that some neurons showed [Ca^2+^]_i_ responses in the micromolar range. Fura-2 is a high affinity Ca^2+^ probe, a technical limitation that leads to underestimating peak [Ca^2+^]_i_ rises for values above 1 μM (Hyrc et al., 1997). We therefore repeated the same set of experiments with the low affinity Ca^2+^ indicator fluo-4FF, a probe that, with a *K*_*d*_ of 9.7 μM, is suitable for the detection of [Ca^2+^]_i_ changes occurring in the 1 μM to 1 mM range. Fluo-4FF experiments confirmed that NMDAR-dependent [Ca^2+^]_i_ increases are identical in nNOS(+) and (−) neurons (Figures 2A–E). Of note, different from fura-2 experiments, in the fluo-4FF data set we observed a decreased recovery time in all neurons, a phenomenon that likely reflects the lower affinity of the probe for Ca^2+^.

Finally, we assessed whether nNOS(+) neurons show differences in [Ca^2+^]_i_ response via other glutamatergic ionotropic receptors (i.e., AMPARs). To that aim, fura-2 loaded striatal neurons were exposed to kainate (50 μM) and [Ca^2+^]_i_ levels analyzed in terms of agonist-evoked peak levels as well as overall cytosolic cation loads (as indicated by evaluation of the area under the curve of fura-2 signals). Even in this set of experiments, we did not observe significant differences between nNOS(+) and (−) striatal neurons (data not shown).

In summary, Ca^2+^ imaging experiments indicated that nNOS(+) neurons possess fully functional NMDARs.

### ROS generation upon NMDA exposure in nNOS(+) and (−) neurons

Aberrant Ca^2+^ entry through NMDARs results in cytosolic Ca^2+^ overload, dissipation of the mitochondrial inner membrane potential, opening of the permeability transition pore, and production of mt-ROS (Rizzuto et al., 2012). ROS are major contributors to excitotoxicity (Choi, 1992a,b; Dugan and Choi, 1994; Sayre et al., 2008). As mentioned above, NADPH diaphorase is the neuronal nNOS enzyme (Hope et al., 1991). Thus, nNOS(+) neurons are likely to be chronically exposed to an intracellular environment that is constantly confronted (and needs to be equipped to deal) with the presence of high NO levels.

To investigate mt-ROS levels generated by NMDAR activation, striatal neurons were first loaded with the ROS sensitive probe DHR (Henderson and Chappell, 1993) and fluorescent changes evaluated before, during, and after NMDA exposure with confocal microscopy. Native DHR is uncharged, not fluorescent, and passively diffuses through membranes. Once in the presence of ROS, DHR is oxidized to the cationic fluorescent product, rhodamine 123, allowing the investigation of mitochondrial ROS production (Dugan et al., 1995).

**Figure 3:**
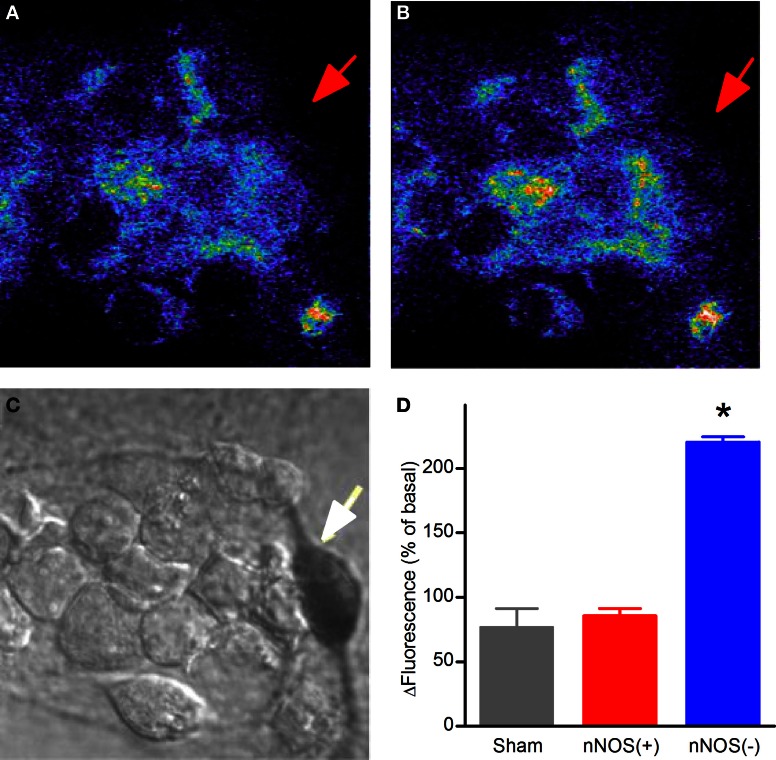
**NMDAR-dependent mitochondrial ROS production in striatal cultured neurons. (A)** Confocal images of basal levels of fluorescence of DHR-loaded neurons prior to NMDA stimulation. **(B)** DHR fluorescence 25 min after a 5 min NMDA exposure. **(C)** Bright field image showing stained nNOS(+), indicated by arrow, and nNOS(−) neurons. **(D)** Quantification of DHR fluorescence increases 25 min after NMDA exposure (^*^p < 0.001). Results are expressed as mean % of *F*_*x*_/*F*_0_ ± SEM; nNOS(+) *n* = 19; nNOS(−) *n* = 142 from 18 independent experiments.

In this set of experiments DHR-loaded neurons, after acquisition of baseline fluorescence levels, were exposed to NMDA (50 + 10 μM glycine) for 5 min, NMDAR activation was then halted by addition of the receptor antagonist (MK-801; 10 μM) and fluorescence changes evaluated up to 30 min. Confocal DHR imaging revealed that NMDA application failed to promote significant fluorescence changes in nNOS(+) neurons while (−) neurons showed significant signal rises (Figure 3).

Thus, DHR experiments revealed that NMDA exposure fails to elicit production of mt-ROS in nNOS(+) striatal neurons.

## Discussion

nNOS(+) neurons are spared from NMDAR-driven excitotoxicity, a phenomenon that has puzzled the field for many years (Beal et al., 1986; Koh and Choi, 1988; Kumar, 2004; El Ghazi et al., 2012). Our study offers two major findings that may help to unravel the mechanisms underlying the decreased vulnerability of these neurons and offer additional insight for their increased survival in the context of HD.

Firstly, nNOS(+) striatal neurons possess fully functional NMDARs and respond to receptor activation with [Ca^2+^]_i_ rises that do not differ for amplitude and temporal dynamics to the ones observed in the overall population of nNOS(−) neurons. Secondly, in nNOS(+) neurons, NMDAR activation does not lead to generation of mt-ROS, thereby occluding a critical downstream event of their excitotoxic cascade.

NMDAR-dependent deregulation of intraneuronal [Ca^2+^]_i_ levels upon prolonged or excessive glutamate exposure is the event that starts excitotoxicity (Choi, 1992a,b, 2005). Thus, the reduced vulnerability of nNOS(+) neurons to excitotoxic challenges could have been easily explained by reduced expression or functioning of NMDARs in this subpopulation.

Our data lend us to hypothesize an alternative view and finally demonstrate that these neurons are most likely resistant not because they differ in number or functionality of NMDARs but, on the contrary, because they are able to set in motion protective events acting downstream to block a key step in the injurious cascade set in motion by NMDAR activation: the intraneuronal raise of mt-ROS levels.

ROS play important roles in several biological functions and are critical mediators of physiological or death signaling (Huang and McNamara, 2012; Ray et al., 2012). Upon excitotoxic conditions, NMDAR-driven production of high levels of cytosolic and mitochondrial ROS as well as nitrosative species leads to neuronal death (Floyd, 1999; Droge, 2002; Lau and Tymianski, 2010). Neurons maintain a redox homeostasis and counteract these oxidative and nitrosative hits by employing several endogenous scavenging mechanisms (Greenlund et al., 1995) like superoxide dismutases, catalases, and glutathione peroxidases.

As nNOS(+) neurons express high levels of nNOS (Hope et al., 1991) and are thereby producing large amounts of NO, it is conceivable that they are primed to deal with an oxidizing intracellular environment and possess an enhanced capability to neutralize this challenge.

Landmark findings support this idea and, in fact, indicate that nNOS(+) express high levels of manganese superoxide dismutase (MnSOD) (Gonzalez-Zulueta et al., 1998). MnSOD, a mitochondrial enzyme, neutralizes free radicals. The enzyme confers resistance against NMDA- and NO-mediated toxicity both *in vivo* and *in vitro* by preventing the generation of toxic peroxynitrite originating from the NO and O^−^_2_ interaction (Beckman and Koppenol, 1996; Gonzalez-Zulueta et al., 1998; Brown, 2010). Thus, our data suggest a conceptual framework in which enhanced MnSOD activity in nNOS(+) neurons might lead to the reduced ROS generation that we find upon NMDA exposure and provides potential explanation for their decreased vulnerability to NMDAR-mediated neuronal death.

Our results are providing support for the idea that mitochondria play a strategic role in the phenomenon. We acknowledge that future experiments are needed to clarify whether increased MnSOD activity is indeed play a major role in the neuroprotective pathway. We show that NMDA exposure produces [Ca^2+^]_i_ levels that are similar in nNOS(+) and (−) striatal neurons. We also show that these excitotoxic Ca^2+^ rises fail to promote significant ROS raises in the mitochondria of nNOS(+) neurons. Thus, in nNOS(+) neurons, the behavior of mitochondria, critical actors of the excitotoxic cascade, differ.

Two major arguments support this assumption. In the Ca^2+^ imaging experiments we have shown that nNOS(+) neurons face the same NMDAR-driven Ca^2+^ overload. If one has to fit these data and the DHR results in a comprehensive conceptual framework, it can be hypothesized that either: (1) mitochondria of nNOS(+) neurons are allowing less Ca^2+^ uptake (thereby decreasing the overall generation of Ca^2+^-dependent mt-ROS) or (2) nNOS(+) neurons have developed ways to counteract the generation of mt-ROS.

When considering the first hypothesis, it should be underlined that our Ca^2+^ imaging experiments take in account and report changes of cation levels occurring in the cytosol, a phenomenon that is the net result of simultaneous and concerted activities of many Ca^2+^ homeostatic systems (Pizzo et al., 2012; Rizzuto et al., 2012). The fact that we observed similar NMDAR-driven Ca^2+^ loads in the two neuronal subpopulations could, in theory, be explained by compensatory mechanisms occurring in nNOS(+) neurons that prevent Ca^2+^ overloads in mitochondria. For this mechanism to work, one has to infer that these cells may have enhanced expression and/or functioning of the plasma-membrane Ca^2+^-ATPases (PMCA), a key pathway for Ca^2+^ extrusion, a possibility that we have not tested yet. Differences in activity of the other major cellular system for Ca^2+^ extrusion, the plasmatic Na^+^-Ca^2+^ exchanger (NCX), are unlikely as excitotoxic conditions (like the one used in our setting) favor either a NCX reverse operational mode (thereby leading the exchanger to serve as pathway for Ca^2+^ entry) (Orrenius et al., 2003) or NCX functional blockade by calpain-mediated cleavage of the exchanger (Bano et al., 2005).

Enhanced functioning of intracellular Ca^2+^ stores (i.e., the endoplasmic reticulum; ER) is also unlikely to work to decrease Ca^2+^ rises in nNOS(+) neurons. Accordingly to the “hot spot” hypothesis proposed by Rizzuto et al. (1998), Ca^2+^ overload in the ER is a detrimental source for ROS generation as the cation eventually exits the ER and is taken up by mitochondria located in the ER vicinity, thereby providing the driving force for production of mt-ROS. Thus, if nNOS(+) neurons do overdrive the ER to maintain Ca^2+^ homeostasis such compensatory mechanism should be counterbalanced by enhanced levels of mt-ROS, the opposite of what we observed in our nNOS(+) neurons.

The idea that mitochondria of nNOS(+) sequester equal amounts of Ca^2+^ but respond to this hit with a decreased generation of ROS is strongly suggested by previous findings (Gonzalez-Zulueta et al., 1998) that indicate a major role for MnSOD in promoting protection against NMDAR-mediated oxidative stress in nNOS(+) neurons. Within this framework, we do speculate that the observed reduction of ROS levels in nNOS(+) neurons may be due to higher scavenging capabilities of this neuronal subpopulation.

In summary, our data offer complementary data that substantiate a major role for mitochondria in promoting the reduced vulnerability to NMDA in nNOS(+) striatal neurons. Given the role played by polyQ Htt in affecting mitochondrial functioning, this mechanism can be particularly relevant in the context of the neuronal loss occurring in the striatum of HD patients and provide targets for therapeutic intervention.

### Conflict of interest statement

The authors declare that the research was conducted in the absence of any commercial or financial relationships that could be construed as a potential conflict of interest.
